# Primary health care nurses attitude towards people with severe mental disorders in Addis Ababa, Ethiopia: a cross sectional study

**DOI:** 10.1186/s13033-019-0283-x

**Published:** 2019-04-11

**Authors:** Yoseph Sahile, Sewbesew Yitayih, Berhanu Yeshanew, Daniel Ayelegne, Awoke Mihiretu

**Affiliations:** 1 Amanuel Mental Specialized Hospital, Addis Ababa, Ethiopia; 2 Department of Psychiatry, School of medicine, College of Medicine and Health Sciences, University of Gondar, Gondar, Ethiopia; 3 Department of Psychiatry, College of Medicine and Health Science, Dire Dawa University, Dire Dawa, Ethiopia; 4 Department of Community Health Nursing, School of Nursing, College of Medicine and Health Sciences, University of Gondar, Gondar, Ethiopia; 5 Department of Psychiatry, Addis Ababa University, College of Health Sciences, School of Medicine Addis Ababa, Addis Ababa, Ethiopia

**Keywords:** Attitude of nurses, Severe mental illness, Nurses, Health center, Primary health care, Addis Ababa, Ethiopia

## Abstract

**Background:**

Negative attitude and discriminatory behavior of health professionals constitute a major obstacle in psychiatric care and have been pointed out as a key issue in working with mental illness. Understanding about the attitude of nurses is crucial for quality and holistic care of psychiatric services and essential for the successful integration of mental health into primary health care. However, there is a paucity of study to examine the attitude of primary health care nurses towards severe mental disorder in Ethiopia. Therefore, this study aimed to assess the attitude of primary health care nurses and its associated factors towards people with severe mental illness in Addis Ababa.

**Methods:**

Institutional based cross-sectional study was conducted among nurses working at primary health care in Addis Ababa from May to June, 2018. Multistage sampling technique was used to select 634 participants. A structured self-administered questionnaire was used. Data were coded and entered into EPIDATA 3.1 and exported to SPSS version 20 for analysis. Bivariate and multivariate binary logistic regression analysis was used to identify factors associated with attitudes of nurses in primary health care. The level of significance was declared at P-value < 0.05 with 95% confidence interval.

**Results:**

A total of 610 respondents were included in the study with a response rate of 96.2%. The mean age of participants was 28.6 ± 5.9 (SD) years and the prevalence of negative attitude was 48.2%. Multiple logistic regression models revealed that respondents who have diploma [AOR = 3.09, CI (1.20–7.95)], work experience of < 5 years [AOR = 4.49, CI (2.37–8.49)], respondents who didn’t took mh-Gap training [AOR = 4.92, CI (3.05–7.95)] and poor knowledge about mental illness [AOR = 2.84, CI (1.82–4.44) were associated with negative attitude towards people with severe mental illness.

**Conclusion:**

Nearly half of the participants have negative attitude towards people with severe mental disorders. Therefore, evidence based and contextualized models are warranted to mitigate negative attitudes of primary health care nurses.

## Introduction

Severe mental disorder (SMD) is explained by a significant disturbance in an individual’s cognition, emotion, or behavior that reflects a dysfunction in the psychological, biological, or developmental processes underlying mental functioning [1]. Currently, SMD is responsible for 12% of the global burden of disease and expected to reach 15% by the year 2020 [2]. Mental illness in Ethiopia is the leading non-communicable disorder in terms of burden that comprised 11% of the total burden of disease [3]. Schizophrenia and depression included in the top ten most burdensome conditions out-ranking HIV/AIDS [3]. Considering the prevalence of the problem, currently mental health has taking the world’s attention. However, the negative attitudes, stigmatization, and discrimination associated with mental illness are an important health issue and broadly explained by different segments of the population [4, 5].

There were reports of stigmatizing behaviors from health care workers towards patients with SMD which includes offering discouraging advice, negative remarks, rejecting behavior, and negative attitudes [6, 7]. Taking this in account, the attitudes and knowledge of nurses on mental illness have been argued to be a major determinant of the quality and inclusive care for people with mental illness [8].

There is high prevalence of nurse’s attitude regarding patients with severe mental disorder across the globe such as Switzerland (55.2%), Jamaica (61%), Nigeria (53%), Zimbabwe (75.6%), Tanzania (58.9%) and Kenya (75%) [8–12]. In Ethiopia, the prevalence ranges from 27 to 57% [13, 14] but the report was about general nurses attitude rather than primary health care workers.

The most frequently reported associated factors for negative attitude of mental health professionals towards people with SMD were being male, have less psychiatric nursing training, and hold junior positions tend to express less favorable attitudes towards people with mental illness [15, 16]. Primary health care nurses with less training, minor exposure and experience in mental health has also reported negative, intolerant and fearful attitudes, and perceptions towards mental illness and mentally ill people [16, 17].

**Fig. 1:**
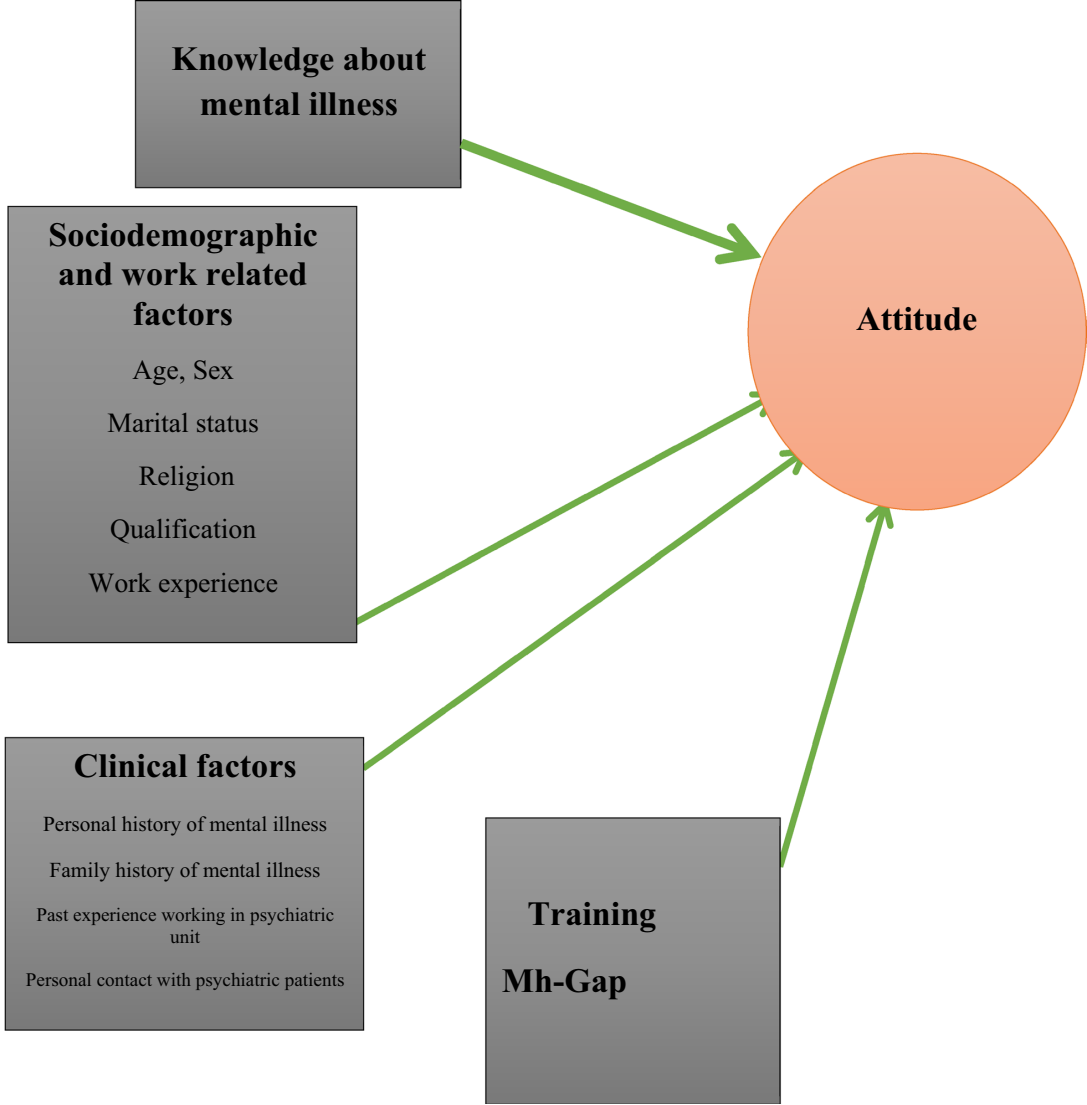
Conceptual frame work for factors affecting attitude of nurses in primary health care in Addis Ababa Ethiopia; 2018

From our experience, in Ethiopia, there seems a clear commitment for improving mental health care and increasing coverage at the highest governmental level. Primary health cares are expected for providing ‘essential health care’ which is universally accessible to individuals and families in the community and provide as close as possible to where people live and work. Implementation of the integration of mental health services at primary health care level was started in 2014. In order to make the integration effective, primary care health professionals were selected to be the key personnel. A study from Nigeria suggests that the major challenges of successful integration of mental health into PHC could be a negative and stigmatizing attitude [18] but there is very limited information in the context of Ethiopia but understanding of the attitude of these professionals is extremely important for the delivery and uptake of mental health services in primary care level. This study will offer a formative information the perception of primary health care nurses’ about people with severe mental illness that may be helpful in designing appropriate training or re-training programs in Ethiopia. The finding will also help to facilitate the integration of mental health service to primary health care level. Thus, we aimed to explore primary health care nurses’ attitude towards people with severe mental disorders (Fig. 1).

## Methods and materials

### Study area, design, and population

Institution based cross sectional study was conducted from May to June, 2018. The study was conducted in Addis Ababa, the capital of Ethiopia. The city has a total of 608 health facilities including hospitals, health centers, health stations and clinics. There are 94 health centers under administrative city. Ministry of health has four tier health care systems and primary health care unit (PHCU) is the 4th tier which is near to the community. This health facility is selected as a ministry of health pilot district for integration of mental health in urban health extension package.

### Source and study population

All nurses who are working at government financed health centers in Addis Ababa were considered as source population. Participants who were available during data collection period were the study participants.

### Sampling technique and procedure

The minimum number of sample required for this study is determined by using single population proportion formula considering the following assumptions:

$${\text{n }} = \frac{{\left( {{\text{Z}}\upalpha /2} \right)^{2} \;{\text{p }}(1 - {\text{p}})}}{{{\text{d}}^{2} }}$$

where n_*i*_= minimum sample size required for the study, Z = standard normal distribution (Z = 1.96) with confidence interval of 95% and α = 0.05, P = proportion of attitudes in nurses in primary health care towards severe mental illness is unknown in our country; so I have used, P = 50% (0.5), d = Absolute precision or tolerable margin of error (d) = 5% = 0.05

$${\text{n}}_{\text{i}} = \frac{{\left( {{\text{Z}}\upalpha / 2} \right)^{ 2}\;{\text{p}}\left( { 1- {\text{p}}} \right)}}{{{\text{d}}^{ 2} }} = \frac{{\left( { 1. 9 6} \right)^{ 2} \times 0. 5\left( { 1- 0. 5} \right)}}{{\left( {0.0 5} \right)^{ 2} }} = 384$$

So, n = 384 because of multistage sampling technique the calculated sample size was multiplied by 1.5. Therefore, n = 384 * 1.5 = 576, adding 10% non-respondent rate the final sample size was = 634.

Thirty percent of the total health centers were selected to take adequate number of health centers to represent the source populations. When calculating 30% of the total HCs, 28 HCs was included in this study. Out of 94 HC 28 HC was selected by using simple random sampling method from lists of all HC and simple random sampling technique was used to select the study subjects and study participants were proportionally allocated to selected health centers.

$${\text{n}}_{\text{x}} = \frac{{{\text{Nx}} \times {\text{n}}}}{\text{N}}$$

where, n = sample size, n_x_ = sample size in each selected health center, N = number of source population, N_x_ = population size in each selected health center, x = number of health centers (1,2,3,4…28)

$$n = n_{1} + n_{2} + n_{3} + n_{4} + n_{5} + n_{6} = 6 3 4$$

### Operational definitions

*Severe mental disorders* According to global mental health definition severe mental disorders mainly includes schizophrenia, bipolar and major depression disorders.

*Negative attitude* greater than (> 57) from MICA-4 scores.

*Mh-GAP training* WHO designed training for scaling up services for priority mental, neurological and substance use disorders.

*Knowledge* awareness about mental illness as measured by knowledge about mental illness questionnaire, National Institute of Mental Health and Neurosciences (NIMHANS), Bangalore, department of psychiatry a modified version for health workers [19].

### Inclusion and exclusion criteria

All nurses who were working in the selected health centers during data collection period were included in the study. Psychiatric nurses were excluded from the study.

### Data collection tools and procedures

An adapted structured questionnaire was used to collect data about socio-demographic characteristics. Knowledge about mental illness was assessed by 13 item tool developed by National Institute of Mental Health and Neurosciences (NIMHANS), Bangalore, department of psychiatry a modified version for health workers [19].

Clinicians’ Attitude Scale (MICA-4), 16 item tool, was used to assess PHC nurses attitude towards people with severe mental illness. The tool was developed to assess attitudes towards severe mental illness of students or staffs in any health discipline. It is a 6-point Likert scale (‘‘strongly agree, agree, somewhat agree, somewhat disagree, disagree and strongly disagree’’). A single overall score is calculated by summing each individual item where a high overall score indicates more negative stigmatizing attitude with a possible range of 16–96 [20]. For the purpose of this study, categorization was done using the mean score. Subjects were categorized as having negative attitude who scores greater than or equal to mean (≥ 58). We did pretest of the instruments and experts were consulted about the content, face and technical validities. Internal consistency of MICA-4 was 0.74 using Cronbach’s alpha.

The principal investigator or the research assistant will invite participants for the study. After reading the information sheet and the consent form, data were collected from those participants who gave consent.

### Data quality assurance

The translated Amharic version of self-administered questionnaire was disseminated to participants. Pre-test with 5% of the total sample size was done before the start of actual data collection at health centers out of the selected area. Minor language revision was made based on the findings of the pre-test. The filled questionnaire was checked daily by the principal investigator for completeness and neatness. Data collectors and supervisors were trained before data collection. The data collectors were psychiatry nurses and supervisors were MSc psychiatry professionals.

### Data processing and analysis

Data was coded and entered in-to EPIDATA 3.1 for cleaning, storing, and recording. The data were exported to SPSS version 20 for analysis. Descriptive statistics (frequencies, percentages, cross tabulations) were used to summarize the sociodemographic and other preliminary data. Bivariate and multivariate binary logistic regression analysis was used to identify factors associated with attitude of nurses in primary health care. The level of significance was declared at P-value < 0.05 with 95% confidence interval.

### Ethical considerations

Ethical clearance was obtained from University of Gondar institutional review board, Amanuel Mental Specialized Hospital ethical review committee and Addis Ababa health bureau Ethical review committee (ERC). Written Informed consent was obtained from each participant during data collection. All participants were informed about the aim and purpose of the study. Study participants were given the right to refuse or withdraw from participation at any time during data collection. All personal information was kept entirely confidential.

## Results

### Socio-demographic characteristics of respondents

The response rate was 610 (96.2%), out of 634 study participants. Four hundred thirty (70.5%) of the study participants were female. The mean age of participants was 28.6 ± 5.9 (SD) years with range of 20 and 55 years. Majority of the respondents, 440 (72.0%) were in the age category of 20–29 years. Among the total participants 408 (66.9%) were orthodox Christian religion followers, 342 (56.1%) were single, 344 (56.1%) have diploma by qualification and 440 (72.1%) of the respondents have < 5 years work experience (Table 1).

**Table 1:** Socio-demographic characteristics of primary health care nurses in Addis Ababa, 2018

Variables	Category	Frequency	Percent
Sex	Male	180	29.5
	Female	430	70.5
Age	20–29	440	72.0
	30–39	134	22.0
	40–49	18	3.0
	≥ 50	18	3.0
Religion	Orthodox	408	66.9
	Muslim	100	16.4
	Protestant	96	15.7
	Others^a^	6	1.0
Marital status	Single	342	56.1
	Married	246	40.3
	Others^b^	22	3.6
Educational status	Diploma	344	56.4
	BSc	231	37.9
	MSc	35	5.7
Work experience	≤ 5	440	72.1
	6–10	66	10.8
	≥ 11	104	17.1
Working department	Medical	316	51.8
	MCH	146	23.9
	Emergency	62	10.2
	Surgical	46	7.5
	ART	40	6.6

N.B. ^a^Catholic, Jehovah, Wakifeta, ^b^widowed, divorced

### Mental health training

**Fig. 2:**
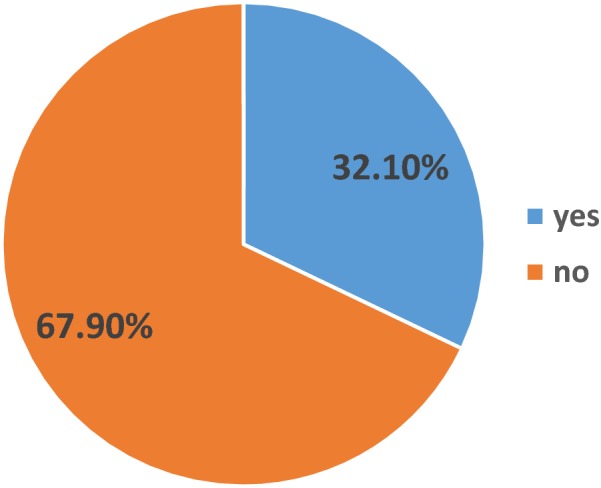
Proportion of Mh-Gap trained primary health care nurses in Addis Ababa, Ethiopia, 2018

Among 610 respondents, 196 (32.1%) of them were trained mh-GAP training (Fig. 2).

### Clinical related factors with nurses’ attitude towards people with severe mental disorders

Related with clinical factors, 60 (9.8%) of the respondents have family history of mental illness. Twenty-four (38.7%) of them have first degree relatives by their relation with the patient (Table 2).

**Table 2:** Clinical factors for attitude and associated factors of primary health care nurses towards people with severe mental illness in Addis Ababa, 2018

Variables	Category			
	Yes	%	No	%
Presence of mental illness in the family	60	9.8	550	90.2
Personal experience of mental illness	74	12.1	536	87.9
Knowing someone with mental illness other than a patient	359	58.9	251	41.1
Work experience at psychiatric unit	65	10.7	545	89.3

### Knowledge of nurses in primary health care about people with severe mental illness

**Fig. 3:**
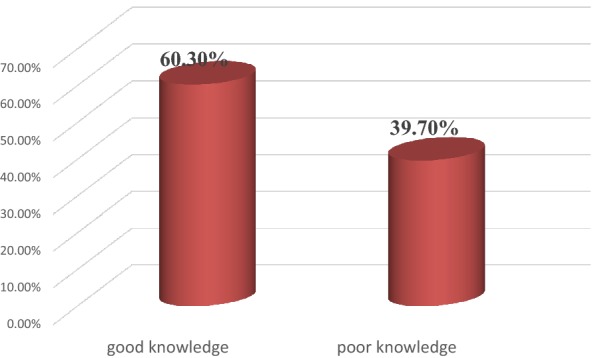
Knowledge of primary health care nurses about people with severe mental illness in Addis Ababa, Ethiopia, 2018

From the total participants of the study, 368 (60.3%) of the respondents have good knowledge about people with severe mental disorders which was evidenced by answering 9 out of 13 knowledge questions correctly (Fig. 3).

### Attitudes of nurses working in primary health care towards people with severe mental illness

**Fig. 4:**
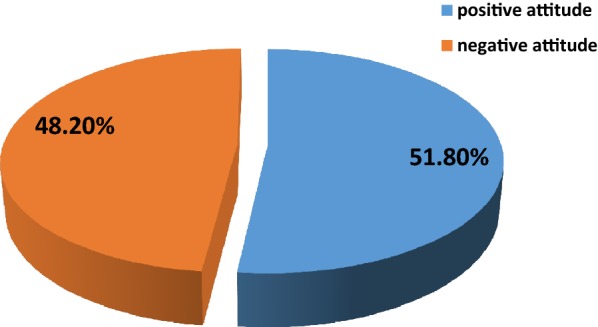
Attitude of primary health care nurses in Addis Ababa, Ethiopia, 2018

Among 610 participants, 294 (48.2%) of nurses have negative attitude towards people with severe mental illness by scoring above the mean (58) of the total score of mental illness clinicians attitude scale (MICAs) (Fig. 4).

### Factors associated with negative attitude

Bi-variate and multivariate analysis was done to test the association between different variables and negative attitude. Variables which were associated (P ≤ 0.25) in the bi-variate analysis was run in the multivariate analysis. As a result of this, the following variables were associated to attitude of nurses: educational status (qualification), work experience, mental health training (mh-GAP) and knowledge about mental illness (Table 3).

**Table 3:** Bi-variate and multivariate factors associated with primary health care nurses attitude towards people with severe mental illness in Addis Ababa 2018 (N = 610)

Variables	Attitude status		COR (95% CI)	AOR (95% CI)	P value
	Positive	Negative			
Educational status					
Diploma	109	235	4.132 (1.98–8.60)	2.96 (1.17–7.48)	0.021
BSc	184	47	0.490 (0.22–1.05)	0.405 (0.16–1.08)	0.07
MSc	23	12	1	1	
Work experience					
≤ 5	172	268	7.44 (4.32–12.8)	4.84 (2.57–9.14)	0.001
6–10	58	8	0.659 (0.269–1.616)	0.24 (0.20–1.50)	
≥ 11	86	18	1	1	
Mental health training (mh-GAP)					
Yes	147	49	1	1	0.001
No	169	245	4.349 (2.980–6.347)	4.95 (3.07–7.96)	
Family history of mental illness					
Yes	48	12	1	1	
No	268	282	1.56 (1.09–2.67)	1.52 (0.74–3.12)	0.25
Personal experience of mental illness					
Yes	55	19	1	1	
No	261	275	3.050 (1.763–5.278)	1.325 (0.617–2.843)	0.47
Previous experience of working in psychiatry unit					
Yes	53	12	1	1	0.07
No	263	282	4.736 (2.475–9.060)	2.319 (0.930–5.782)	
Knowledge about severe mental illness					
Good	240	128	1	1	0.001
Poor	76	166	4.095 (2.898–5.788)	2.83 (1.82–4.39)	

The odds of developing negative attitude among nurses’ with educational status of diploma was 3.06 [AOR = 3.06 (95% CI 1.18, 7.89)] times higher as compared to participants who hold MSc. The likelihood of having negative attitude among nurses having work experience of < 5 years were 4.53 [AOR = 4.53 (95% CI 2.39–8.56)] times higher as compared to those who have work experience ≥ 11 years. The odds of developing negative attitude among nurses who had no mental health training were about 4.88 [AOR = 4.88 (95% CI 3.02–7.89)] times higher as compared to those participants who had training. The odds of having negative attitude among nurses who have poor knowledge about mental illness were 2.84 [AOR = 2.84, (95% CI 1.82–4.44)] times higher than those who have good knowledge.

## Discussion

This study was the first attempt to ascertain attitude and associated factors towards people with severe mental disorders among nurses who are working in primary health care in cities, Ethiopia. The current study indicated relatively high prevalence of negative attitude among primary health care nurses towards people with severe mental disorders. The current finding was consistent with in a study conducted in other high and low resources settings such as Jamaica, Switzerland, Malaysia, and Greece [21–23]. The prevalence of attitude of nurses towards people with SMD was very high in African settings such as in Kenya, Tanzania, Zambia, South Africa and Nigeria. This implies that negative attitude of primary health care nurses towards people with SMD is a global problem [6, 10, 11, 24]. Similar explanatory model of mental illness and poor prognosis of the disorder across different settings might be the reasons. In some cultures nurses might explain supernatural causes for SMD and in settings where there is no effective interventions, in Africa, negative attitude might be higher.

The different in psychometric properties of different instruments across different settings might revealed different prevalent outcomes. To mention few of the instruments, opinion about mental illness scale, attitude scale for mental illness (ASMI), attitude scale for mental illness (ASMI) and community attitude towards mental illness scale were used in Kenya, South Africa, Zambia and Nigeria respectively.

Against the above findings, positive attitude towards people with severe mental disorders were reported from Bhutan, India, and Sweden among mental health nurses [23, 25, 26]. The difference with the current study and previous reports might be due to differences in that the theoretical training, increased interpersonal contact with people with mental illness, education and clinical experience in mental health which are important indicators for reduced negative attitude towards people with mental disorders.

In Ethiopia, slightly different prevalence reports have been reported from hospital nurses [13, 14]. Slight differences could be explained by the use of different measures of outcome. Of course, availability of training and exposure to the treatment of people with SMD could contribute for outcome differences.

Professional qualifications (nurses who have diploma) were 2.96 times more likely to have negative attitude than those with educational level of MSc. This is supported by study in Ethiopia among nurses working in public hospitals and study done in South Africa Durban among general nurses [13, 27]. This finding reveal that nurses with higher educational level will have less stigmatizing attitude than those with lower level of educational status, and professionals with increased academic level will have the opportunity to have more theoretical knowledge about mental illness and the chance to have frequent contact with individuals with mental illness [27].

According to the current study, primary health care nurse with work experience of less than 5 years were 4.84 times more likely to have negative attitude than nurses with work experience of more than 11 years. This study is supported by studies from other settings in Ethiopia and abroad, South Africa and Nigeria [13, 28]. But this finding is different from a study from Bhutan among general nurses which implies that nurses with work experience of more than 20 years were significantly associated with negative attitudes than who have less clinical experience. This might be due to inadequate education especially with regard to working with people with serious mental disorder and the instrument was designed to assess the attitude of the community rather than clinicians attitude [27].

Regarding to mental health training nurses who didn’t take mental health training was more likely to show negative attitude than those who had training by 4.95 times. This is in line with studies done in Taiwan, Republic of Ireland and Finland where nurses with less mental health training endorsed negative and stigmatizing attitude for people with severe mental disorders [15, 17]. This is known that health training, clinical experience and increased interpersonal contact with people with mental disorders would reduce negative and stigmatizing attitude [6]. The odds of having negative attitude among nurses with poor knowledge about mental illness were 2.83 times higher as compared to nurses with good knowledge. This aligns with the findings in the WHO report and a study conducted in Sweden [8, 29] which could be explained by lack of adequate training and less supervision by mental health teams as a cause of lack of knowledge. Accordingly, it might be argued that increased level of training have the effect of bringing about a decrease in negative attitude among nurses concerning people with mental disorders and this would reinforce the impression that increased level of knowledge about mental illness has a direct bearing on attitude development among the nurses [30].

## Conclusion

About half of the participants have negative attitude towards people with severe mental disorders. Educational status, mental health training, professional experience and knowledge about mental illness were significantly associated with negative attitude. Therefore, it is important to re-initiate training programs for the primary health care nurses to reduce negative attitudes towards people with severe mental disorders.

## Abbreviations

AOR: adjusted odds ratio
CAMI: community attitude towards mental illness
CI: confidence interval
COR: crude odds ratio
EPI INFO: epidemiological data
HC: health center
KAP: knowledge, attitude and practice
LMICs: low and middle income countries
PHC: primary health care
SMI: severe mental illness
SPSS: statistical package for social sciences
WHO: World Health Organization
